# Left Bundle Branch Area Pacing with a Defibrillator Lead in a Patient with Recurrent Ventricular Tachycardia and Severe Nonischemic Cardiomyopathy: A Case Report

**DOI:** 10.19102/icrm.2026.17043

**Published:** 2026-04-15

**Authors:** Arindam Pande, Dilip Kumar, Rabin Chakraborty

**Affiliations:** 1 Manipal EM Bypass Hospital, Kolkata, India; 2 Department of Cardiology, Manipal EM Bypass Hospital, Kolkata, India

**Keywords:** Cardiac resynchronization therapy, implantable cardioverter-defibrillator, left bundle branch area pacing, ventricular tachycardia

## Abstract

Left bundle branch area (LBBA) pacing is an innovative approach for permanent cardiac pacing. Combining LBBA pacing with an implantable cardioverter-defibrillator (ICD) system can reduce the number of leads required in patients who need both therapies, thereby lowering hardware burden, reducing costs, and potentially improving safety. In this case report, we describe the successful implantation of an ICD lead for LBBA pacing. To our knowledge, this represents the first documented instance of achieving LBBA pacing with an ICD lead positioned using a manually shaped stylet.

## Introduction

Since the initial demonstration by Huang et al. in 2016, left bundle branch area pacing (LBBAP) has emerged as a significant advancement in achieving synchronized ventricular activation.^1^ LBBAP is now recognized as a therapeutic option for heart failure (HF), providing an alternative to conventional cardiac resynchronization therapy (CRT). It also plays a significant role in both preventing and managing pacemaker-induced cardiomyopathy.^2,3^

Implantable cardioverter-defibrillators (ICDs) remain the cornerstone of therapy for the primary and secondary prevention of sudden cardiac death. A substantial proportion of ICD recipients also require permanent pacing. This need is typically addressed by using defibrillator leads for the dual purpose of pacing and defibrillation without compromising shock efficacy. Historically, right ventricular (RV) apical lead placement has been favored due to its stability, particularly for heavier ICD leads. With the emergence of LBBAP, efforts have been made to integrate this physiological pacing technique with ICD systems. Early approaches involved placing an additional lead in the left bundle branch area (LBBA), connected via either the RV port or the left ventricular (LV) port (using a CRT with defibrillator [CRT-D] generator and the R1 connector). However, this method of simply adding a lead often yielded suboptimal performance and increased hardware burden.^4^

Recent advances have explored using the defibrillator lead itself to achieve LBBAP. In 2023, Huybrechts et al. demonstrated the feasibility of positioning an ICD lead in the LBBA using manually shaped delivery sheaths, reporting a success rate of 60% (3/5 patients).^5^ Subsequently, in 2024, Imnadze et al. reported a series of 12 successful cases employing a pre-shaped delivery sheath (Direct Universal Sheath; Abbott, Chicago, IL, USA).^6^ Additional case reports have since emerged,^7,8^ yet no published literature has described achieving LBBAP by shaping the stylet itself for ICD lead delivery, without the use of a specialized delivery sheath.

We report, to the best of our knowledge, the first documented case of successful LBBAP using a single-coil defibrillator lead (Reliance 4-Front; Boston Scientific, Marlborough, MA, USA) positioned with a manually shaped stylet.

## Case presentation

**Figure 1:**
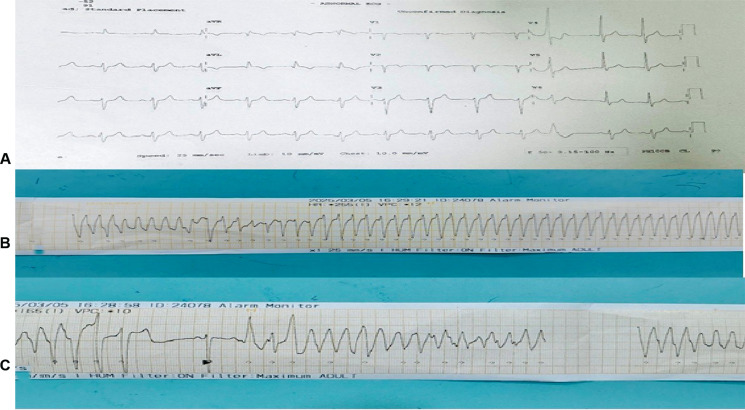
**A:** Baseline electrocardiogram. **B, C:** Ventricular tachycardia.

A 52-year-old man with diabetes mellitus presented with New York Heart Association class III exertional dyspnea that had persisted for 6–8 months. He also reported two episodes of syncope, both with spontaneous recovery. An electrocardiogram (ECG) showed sinus rhythm with left anterior hemiblock, a normal QRS width, and frequent ventricular premature complexes **(Figure 1A)**. Holter monitoring for 24 h was non-contributory. Transthoracic echocardiography (TTE) demonstrated severe systolic dysfunction (LV ejection fraction of <30%) with LV dilation.

During admission for coronary angiography, the patient developed pulseless ventricular tachycardia (VT) **(Figure 1B)** with hemodynamic collapse. This event necessitated emergency measures, including direct current cardioversion **(Figure 1C)**, three cycles of cardiopulmonary resuscitation, endotracheal intubation, and vasopressor support. Post-resuscitation, he required mechanical ventilation and high-dose inotropic therapy. A subsequent episode of VT was managed with additional direct current cardioversion and anti-arrhythmic agents.

Coronary angiography revealed minor, non-obstructive coronary artery disease with codominant circulation. Given the patient’s history of recurrent VT and advanced HF, an automated ICD (AICD) was indicated. However, due to his background of cardiac arrhythmia and advanced HF with reduced ejection fraction, the planned high-dose β-blocker and potential anti-arrhythmic therapy were expected to create significant pacing dependency. This would likely lead to subsequent deterioration of LV function from chronic RV apical pacing.

The potential role of CRT-D implantation was therefore discussed. However, the possibility of a suboptimal or even negative response to mandatory biventricular pacing—given the patient’s normal QRS width—was a significant concern.

Following a multidisciplinary discussion, the decision was made to proceed with dual-chamber AICD implantation, placing the RV lead in the LBBA to achieve physiological pacing, alongside guideline-directed medical therapy for HF.

The authors obtained the required ethical clearance and patient informed consent.

### Procedure details

#### Implantation technique

Under local anesthesia and conscious sedation, dual venous access was established. The left subclavian vein was accessed via an extrathoracic puncture. The His bundle was localized using a catheter advanced from the right femoral vein; this catheter also provided temporary backup pacing.

**Figure 2:**
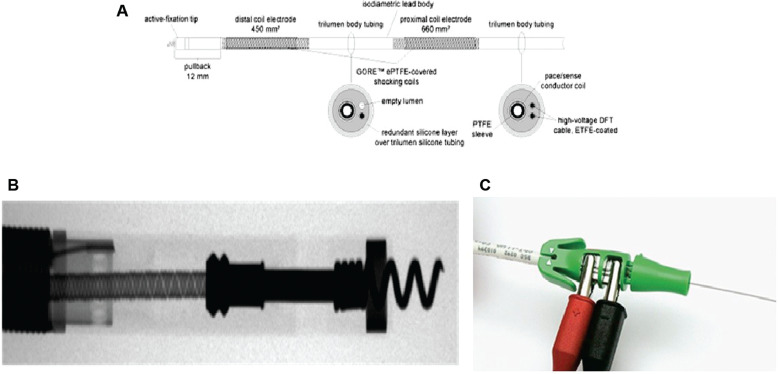
**A:** Basic architecture of Reliance 4-Front implantable cardioverter-defibrillator lead.^9^ Reliance 4-Front lead with extended helix^9^ **(B)** and EZ-4 connector **(C)**.^9^ *Abbreviations:* DFT, defibrillation testing; ETFE, ethylene tetrafluoroethylene; PTFE, polytetrafluoroethylene.

A single-coil ICD lead (Reliance 4-Front, 7.3 Fr, 2.4 mm) was advanced into the RV. The stylet was withdrawn, manually reshaped to avoid sharp bends and prevent hinge-point damage, and then reintroduced into the lead **(Figure 2A)**. Stylet reshaping was done to create a large smooth curve in a single plane over the distal 15 cm followed by an additional posterior 90° curve in the distal most 3 cm, similar to modified Mond’s curve to reach the RV mid-septum. Using the His catheter tip as an anatomical guide, the ICD lead was maneuvered into the LBBA. Electrocardiographic mapping via the ICD lead’s tip confirmed LBBA localization prior to helix deployment **(Figure 2B)**.^9^

#### Lead fixation and testing

**Figure 3:**
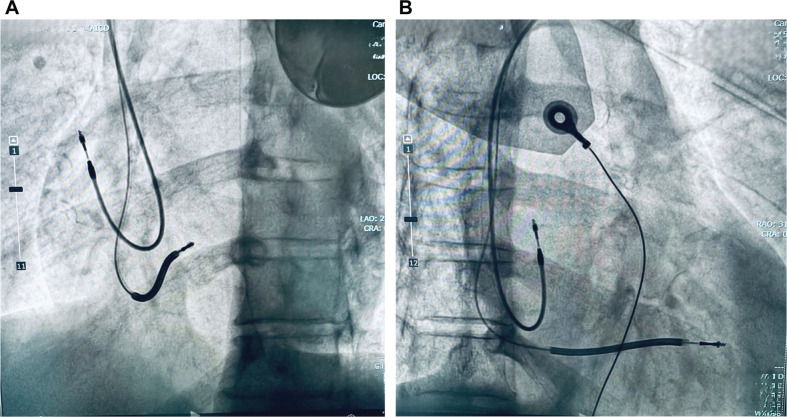
Left anterior oblique **A:** and right anterior oblique **B:** views of the final lead position.

At the target site, septal penetration was done once the lead was in contact with the septum, and the helix was extended using an EZ-4 connector tool (Boston Scientific) with slight over-rotation (16–18 clockwise turns; the manufacturer recommends 11) **(Figure 2C)**. The heavy nature of the ICD lead facilitated penetration of the interventricular septum to reach the LV sub-endocardium—a maneuver not feasible with a conventional pacing lead without the support of a delivery sheath. Fluoroscopy in the right anterior oblique and left anterior oblique views confirmed proper lead positioning, and radiographic assessment of the distal coil was performed to exclude hinge-point damage **(Figures 3A and 3B)**. LBBAP was confirmed by the presence of an r′ deflection in lead V1 during deep septal pacing from the implanted shock lead. Perforation into the LV cavity was ruled out by the absence of a sudden drop in lead impedance.

Electrical parameters were as follows:

- Current of injury: 40 mV
- Threshold: 0.8 V @ 0.5 ms
- Impedance: 765 Ω

**Figure 4:**
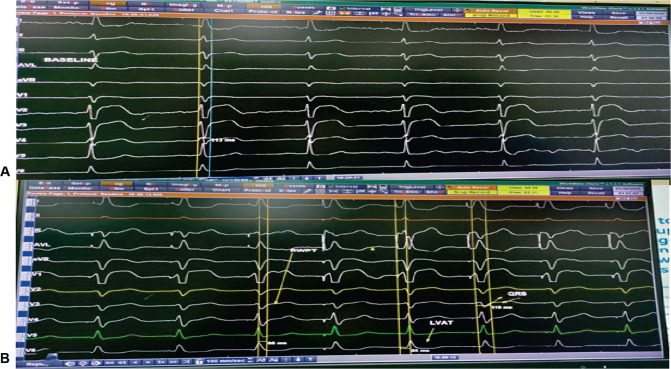
Left bundle branch area pacing parameters. **A:** Intracardiac electrocardiogram showing baseline QRS duration of 113 ms. **B:** At high-output (5V) unipolar pacing, the left ventricular activation time (LVAT) was 78 ms, as shown in the figure; at a lower output (1V), there was the appearance of an Rsr’ pattern in V1, though the LVAT remained same, suggesting non-selective to selective left bundle branch capture..

Electrocardiographic criteria included an rSR′ pattern in V1, an LV activation time of 78 ms, and a QRS duration of 106 ms **(Figures 4A and 4B)**.

**Figure 5:**
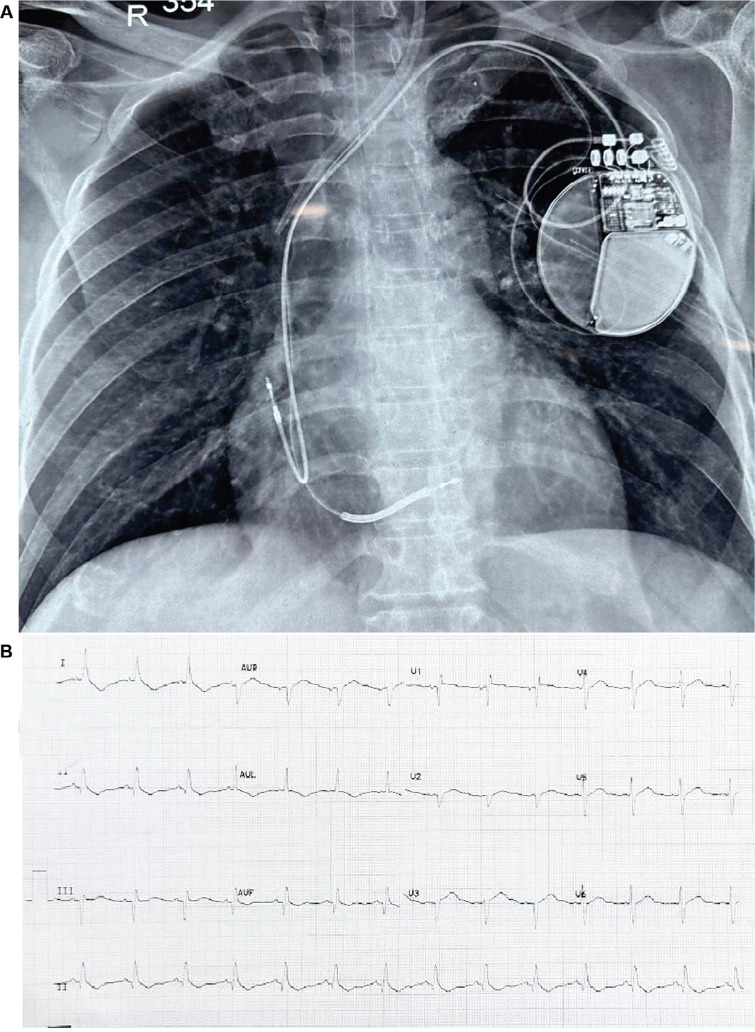
**A:** Post-procedure chest X-ray showing lead positions. **B:** Post-procedure electrocardiogram.

A standard atrial lead was implanted in the right atrial appendage and connected to the ICD generator (using an RF-4 connector; Boston Scientific). Defibrillation testing was performed successfully without lead dislodgement or significant changes in lead parameters post-shock. The pocket was closed in layers. A post-procedure chest radiograph and ECG are shown in **Figures 5A and 5B.**

The patient was discharged home after two additional days in the hospital. The first follow-up was performed 7 days later. Two months post-procedure, the patient received a shock and presented to the hospital for evaluation. Interrogation of the AICD revealed an appropriate defibrillation shock for VT/ventricular fibrillation (VF), which was delivered after anti-tachycardia pacing failed to terminate the arrhythmia.

## Discussion

To the best of our knowledge, we present the first reported case of successful LBBAP achieved using a single-coil ICD lead (Reliance 4-Front) positioned with a manually shaped stylet. The lack of availability of pre-shaped, dedicated delivery sheaths prompted this innovative approach. The lead demonstrated satisfactory sensing, pacing, and defibrillation capabilities, including accurate detection and termination of induced VF.

### Comparison with prior techniques

Recent studies have explored LBBAP integration with ICD systems, albeit with certain limitations. Clementy et al. achieved VF detection through an LBBAP lead connected to the RV port; however, their approach still required a separate ICD lead for shock delivery, thereby increasing the hardware burden.^10^ Huybrechts et al. successfully implanted the Reliance 4-Front lead in three of five patients using customized 8.5-Fr sheaths (Swartz™; Abbott), though the procedure was complex and required guidance with transesophageal echocardiography and general anesthesia.^5^ Conversely, Imnadze et al. reported a high success rate (92%, 11/12 patients) using a pre-shaped 10-Fr delivery sheath (Abbott) for a 6.8-Fr Durata ICD lead.^6^

In contrast, our approach used a manually shaped stylet without the need for specialized sheaths to achieve successful LBBA positioning. This demonstrates the feasibility of performing the procedure using standard tools in experienced hands. Post-defibrillation testing confirmed stable lead parameters, with no evidence of macro-dislocation or significant tricuspid regurgitation on TTE. Furthermore, the successful delivery of an appropriate shock during follow-up confirms the system’s proper functionality in a real-world clinical scenario.

### Technical considerations and future directions

While feasible, LBBA-ICD lead implantation remains technically demanding due to:

#### Material limitations

The lack of dedicated sheaths or leads for this purpose often necessitates improvisation, such as manual stylet reshaping. Currently available delivery sheaths are suboptimal, as their pre-formed curves often lose shape due to the heavy and stiff nature of standard ICD leads. Future development of dedicated tools should focus on three-dimensional, pre-shaped sheaths with enhanced support to accommodate the rigidity of ICD lead coils. Alternatively, designing dedicated ICD leads with thinner or more flexible shock coils could prevent the deformation of delivery sheaths.

#### Lead design challenges

Examples include the following:

- *Flexibility.* A more pliable distal coil could enhance deliverability with existing sheaths.
- *Pacing configuration.* Unipolar programming (tip–can) effectively avoids unwanted RV septal capture in LBBAP. While integrated bipolar pacing (tip–distal coil) may be a viable alternative, it requires optimal tip-to-coil spacing. Therefore, the development of future ICD leads dedicated to LBBAP must incorporate specific tip–coil distances. This design is critical to avoid inadvertent RV septal capture during mandatory bipolar ICD pacing, a particular concern in patients with septal hypertrophy.

### Study limitations and long-term uncertainties

This case report cannot comment on long-term lead stability, performance, or safety. Larger studies with dedicated materials (eg, LBBA-optimized sheaths/leads) and extended follow-up are needed to evaluate:

- Chronic thresholds and sensing reliability;
- Lack of safety data using this lead in this manner;
- Risk of late dislodgement or tricuspid valve interference;
- Impact on pacing-induced cardiomyopathy prevention; and
- Uncommon but reported issues affecting integrated bipolar leads when the coil is located close to the atrium, such as atrial oversensing, which may lead to inappropriate shocks or asystole (in patients with atrioventricular block).

Our case highlights the potential of LBBA-ICD lead implantation using conventional tools, offering a promising avenue to unify physiological pacing and defibrillation. However, broader adoption awaits innovations in lead design and delivery systems to improve procedural efficiency and reproducibility.

## Conclusion

This case demonstrates the feasibility of achieving LBBAP and defibrillation by positioning a single-coil ICD lead (Reliance 4-Front) in the LBBA using a manually shaped stylet. The procedure resulted in successful VF detection and termination without acute complications. While this innovative approach offers the potential to unify physiological pacing and ICD therapy, its technical complexity highlights the need for dedicated delivery tools and LBBA-optimized leads to improve reproducibility. Future studies must evaluate long-term lead stability, electrical performance, and clinical outcomes compared to conventional systems, as current experience remains limited to acute procedural success. Until such evidence emerges, this technique represents an investigational yet promising advancement in device therapy for patients requiring combined pacing and defibrillation capabilities.
